# Comparison of electric hand dryers and paper towels for hand hygiene: a critical review of the literature

**DOI:** 10.1111/jam.14796

**Published:** 2020-08-14

**Authors:** K.A. Reynolds, J.D. Sexton, A. Norman, D.J. McClelland

**Affiliations:** ^1^ Zuckerman College of Public Health The University of Arizona Tucson AZ USA

**Keywords:** air dryer, hand dryer, electric dryer, hand hygiene, paper towel

## Abstract

Numerous studies are published on the benefits of electric hand dryers *vs* paper towels (PT) for drying hands after washing. Data are conflicting and lacking key variables needed to assess infection risks. We provide a rapid scoping review on hand‐drying methods relative to hygiene and health risks. Controlled vocabulary terms and keywords were used to search PubMed (1946–2018) and Embase (1947–2018). Multiple researchers independently screened abstracts for relevance using predetermined criteria and created a quality assessment scoring system for relative study comparisons. Of 293 papers, 23 were included in the final analysis. Five studies did not compare multiple methods; however, 2 generally favoured electric dryers (ED); 7 preferred PT; and 9 had mixed or statistically insignificant results (among these, 3 contained scenarios favourable to ED, 4 had results supporting PT, and the remaining studies had broadly conflicting results). Results were mixed among and within studies and many lacked consistent design or statistical analysis. The breadth of data does not favour one method as being more hygienic. However, some authors extended generalizable recommendations without sufficient scientific evidence. The use of tools in quantitative microbial risk assessment is suggested to evaluate health exposure potentials and risks relative to hand‐drying methods. We found no data to support any human health claims associated with hand‐drying methods. Inconclusive and conflicting results represent data gaps preventing the advancement of hand‐drying policy or practice recommendations.

## Introduction

The term hygiene has a complex set of meanings but is generally associated with ‘conditions and practices that help to maintain health and prevent the spread of disease’ (World Health Organizationn.d.). Effective hand hygiene is well recognized as a primary defence against communicable diseases in both clinical and nonclinical environments (Bolon 2016; Zivich *et al*. 2018). Agencies such as the Centers for Disease Control and Prevention (CDC), World Health Organization (WHO) and US Food and Drug Administration (FDA) have published extensive guidelines on recommended wash times and protocols for hand hygiene practices in medical, food preparation and other environments (Boyce and Pittet 2002; World Health Organization 2009; FDA 2017). CDC guidelines suggest scrubbing hands for at least 20 s with soap and water, which has been shown to reduce approximately 1·5 log CFU per hand (Jensen *et al*. 2015). A 30 s wash resulted in a 2·42 log CFU reduction in another study (Todd *et al*. 2010).

In addition to handwashing, effective hand drying is important in reducing the level of bacterial contamination present on hands and available for transfer from skin to other surfaces, including skin, food and fomites. Several studies have shown more efficient transfer of bacteria to surfaces from wet compared to dry hands or in high‐humidity environments (Patrick *et al*. 1997; Todd *et al*. 2010; Lopez *et al*. 2013; Gerba *et al*. 2016; Kimmitt and Redway 2016). Patrick *et al*. (1997) found that hand drying reduced transfer by up to 99·8%.

Numerous studies have been published with the objective to evaluate the ‘best’ method for hand drying, with the two most common categories being electric hand dryers and towels. Electric hand dryers include both warm air and jet air designs, while towel drying includes disposable paper and cloth products. Some studies also evaluated evaporation or wiping of hands on clothing as a means of drying hands.

Variables considered include bacterial removal efficacy, environmental contamination potentials, ecological or cost benefits, noise and overall hygiene comparisons. Reports in the literature are mixed in terms of which method is more hygienic and consensus has not been found across studies as to which method is the most preferred. However, publications in both the peer‐reviewed and grey literature have made recommendations relative to hygiene and health concerns generally favouring disposable towels over dryers despite a lack of evidence or experiments to appropriately evaluate health outcomes. Most studies have been sponsored by industries with potentially biased interests and have been conducted under a wide range of scenarios with inconsistent study design variables, making it difficult to compare study results.

The objective of this paper is to provide a scoping review of the current scientific peer‐reviewed publications comparing the use of paper towels (PT) *vs* electric dryers (ED) and their impact on environmental health using a standardized set of criteria. Our focus was to categorize, and quantitatively prioritize, the papers relative to scientific rigor in the study design and compare statistically significant results across defined categories of hand‐drying methods. We also examined statistically significant results relative to paper conclusions and the impact of these studies on public perception (as determined by media posts and other grey literature) and provided recommendations for future studies.

## Materials and methods

Scoping reviews are a preferred approach to explore emerging research areas, allowing for a broader view of the depth and breadth of available literature (Grant and Booth 2009). A scoping review typically does not utilize a formal quality assessment in the initial screening, allowing for characterization of the quantity and quality of the literature as part of the data extraction process.

In order to determine next steps towards improved exposure and risk assessments relative to hand hygiene practices, we were interested in a rapid (<6 month) assessment of the current body of literature aimed at identifying gaps in existing research (Khalil *et al*. 2016). This approach allowed us to consider a broad range of publication types and furnish as comprehensive an overview of the topic as time allowed. We followed a previously published methodology consisting of five steps: (i) identifying the research question; (ii) identifying relevant studies; (iii) selecting studies; (iv) charting the data; and (v) collating, summarizing and reporting the results (Arksey and O’Malley 2005; Levac *et al*. 2010).

### Identifying the research question

Data relative to the most effective methods for hand hygiene (i.e. handwashing and drying) are highly varied. Many studies have evaluated the efficacy of various soap products and formulation, water temperature, duration and frequency of washing, as well as drying methods. After reading several conflicting reports from the grey literature (i.e. media reports and internet blogs) (Astor 2018; Dean 2018), with some recommending avoidance of air dryers for certain environments (Hafner 2018), we sought to conduct a scoping review of the peer‐reviewed literature to answer the research question: Are hand dryers more hygienic than paper towels? Given the implication that improved hygiene is correlated with health, we also sought to answer the question: Are PT safer than hand dryers relative to human infection risks?

### Identifying relevant studies

Controlled terms (e.g. MeSH and Emtree) and keywords were used to search PubMed (1946–2018) and Embase (1947–2018) (Table 1). Literature searches were completed on 23 October 2018. No limits were applied to the searches (e.g. publication date, language). Relevant items (articles/reports) identified through Google Scholar were also included for screening. Articles published after the initial search date and up to February 2019 were included if they met the inclusion criteria.

**Table 1:** Scoping review search terms

PubMed	Embase
(‘Paper’[Mesh] OR ‘desiccation’[mesh] OR ‘Hand‐drying methods’ OR ‘paper towel’ OR ‘paper towels’ OR ‘hand‐drying’ OR ‘towel drying’ OR ‘paper towel drying’ OR ‘jet air dryer’ OR ‘Dyson’ OR ‘hand‐drying method’ OR ‘hand‐drying methods’ OR ‘warm air dryer’ OR ‘warm air dryers’ OR Airblade OR ‘Rubbing hands’) AND (‘Hand disinfection/methods’[Mesh] OR ‘Hand/virology’[Mesh] OR ‘Hand/microbiology’[mesh] OR ‘fingers/microbiology’[mesh] OR ‘skin/microbiology’[mesh] OR ‘hand hygiene’ OR ‘washed hands’)]	('hand washing'/exp OR 'hand washing' OR 'hygiene'/exp OR 'hygiene' OR 'hand hygiene'/exp OR 'hand hygiene' OR 'washed hands') AND ('hot air hand dryer' OR 'air dryer' OR 'paper towel' OR 'paper towels' OR 'paper towel drying' OR 'jet air dryer' OR 'ultra rapid hand dryer' OR 'conventional warm air hand dryer' OR ‘Dyson’ OR 'hand‐drying method' OR 'hand‐drying methods' OR 'warm air dryer' OR 'warm air hand dryer' OR 'hand‐drying device' OR 'proper drying of hands' OR 'ultra‐rapid hand dryer' OR ‘Airblade’ OR 'rubbing hands' OR 'effective hand drying')
113	175
Searched 23 October 2018	Searched 23 October 2018

Discovered papers were uploaded into Covidence software (Covidence.org), which provided a standard platform for organizing, screening and de‐duping articles. Remaining articles were randomly assigned and reviewed by at least two of our three‐member team (KAR, AN and JDS). Two authors (KAR and JDS) independently screened full‐text articles for relevance. In cases of disagreement, the third author (AN) discussed the criteria with each reviewer and made a final inclusion decision.

Full‐text articles and reports were selected if they met the following inclusion criteria: (i) involved quantitative assessments; (ii) utilized sampling for environmental microbes or tracers; (iii) evaluated one or more hand‐drying methods; and (iv) published in peer‐reviewed literature. Case studies, reviews, systematic reviews and opinion articles were excluded from the quantitative synthesis but may have been included in the summary of grey literature. Articles were not excluded due to poor quality but rather a process of assessment was utilized during data extraction to detail and compare study design and analytical rigor with reported conclusions to identify gaps and future research needs.

### Extraction of results

Results were extracted from individual papers using a standardized questionnaire formatted using the Qualtrics online survey software (Qualtrics.com). Extracted data included: author, year, study design, drying method, tracer, environment, sampling site, results, study duration, major findings, strengths, limitations, country, funding source and other notes/comments.

## Results and discussion

### Search results

The initial search returned 287 studies. Further review led to the identification of 6 more papers of interest, for a total of 293 studies. Of the 229 articles that remained after duplicates were removed, 191 were excluded after title and abstract screening because of irrelevance to the topic. Inclusion criteria, as outlined above, were applied to the full text of 38 articles. Of these, 23 met the full set of criteria and were included in the final review.

### Major study findings

The majority of studies selected for final data extraction included a comparison of multiple drying methods to determine which drying method was the ‘best’. Table 2 provides detailed information on citations, funding, study design and major findings of extracted papers. In most instances, the chosen best method resulted in comparatively less contamination of the environment (surfaces, air, clothing, hands). Papers targeting economic or ecological impacts of drying methods were excluded from the formal data extraction.

**Table 2:** Summaries of included hand drying articles

Citation and Country	Funding source	Study design, drying method	Major findings
Matthews and Newsom (1987), UK	Building Works Directorate, Department of Health and Social Security^†^	Simulation PT; WD	Significantly lower bacterial levels in aerosols with WD compared to PT
Blackmore (1989), UK	Hygiene Higher Ltd.*, Scott Ltd.*, Initial Industrial Services*	Observational PT; CT; WD	WD contributed to significant increase in bacterial load, PT preferable to CT
Ngeow *et al*. (1989), Malaysia	University Hospital, Kuala Lumpur	Simulation PT; WD	WD dispersed bacteria up to 3 feet *vs* no measurable dispersal with PT
Ansari *et al*. (1991), Canada	Not provided	Simulation PT; CT; WD	Rotavirus reduced (91·74 ± 0·81%) when washed hands were dried with WD, PT reduced (86·90 ± 2·42%) and CT (80·40 ± 3·14%)
Hanna *et al*. (1996), Australia	Deakin University^†^; Spotless Hygiene Systems*	Simulation PT; WD; CT	Drying methods removed few bacteria from hands. Wash waters for first wash on all types of drying methods showed similar bacterial removal (12–16%). Second wash showed that air drying was least effective *vs* CT and PT 1·4 *vs* 17% and 22% respectively
Patrick *et al*. (1997), New Zealand	New Zealand Towel Services*	Simulation; Observational CT; WD	CT *vs* WD drying efficiency and bacterial transfer rates to skin are similar: 99 *vs* 97% and 2 *vs* 5% respectively. Recommend combined 10 s CT/ 20 s WD use
Gustafson *et al*. (2000), US	Mayo Foundation for Medical Education and Research*; Green Electrical Supply*	Observational; Intervention; PT; CT; WD; EV	WD ranked best for removal of bacteria from washed hands but data demonstrates no significant difference among drying methods
Taylor *et al*. (2000), UK	Warner Howard Group Ltd*	Simulation; Observational PT; WD	No significant difference in microbial aerosols generated by PT & WD; PT dispenser contamination similar to other washroom surfaces; WD reduced counts of bacteria in lab and washroom environments by up to 75%
Montville *et al*. (2002), US	Rutgers New Jersey Agricultural Experiment Station^†^; Sloan Valve*	Simulation; Risk model PT; WD	WD increased & PT slightly decreased bacterial contamination on hands
Harrison *et al*. (2003), UK	Georgia‐Pacific Health Smart Institute*	Simulation PT	Bacteria transfer rates from contaminated hands to PT dispenser exits and from contaminated PT exits to clean hands ranged from 0·01 to 0·64% and 12·4 to 13·1% respectively
Yamamoto *et al*. (2005), Japan	College of Nursing University of Tsukuba^†^	Simulation PT; WD	WD no rubbing significantly decreased log bacteria on palms fingers and fingertips but increased with rubbing; PT significantly decreased bacteria on fingertips but not palms and fingers
Snelling *et al*. (2011), UK	Dyson*	Simulation PT; WD; JD; EV	Improved drying efficacy of JD *vs* WD resulted in less transfer to surfaces. PT use resulted in the greatest drying and bacteria removal efficacy. Differences were statistically significant in most but not all cases
Gendron *et al*. (2012), Canada	NSERC^†^ and Canadian Institute of Health Research^†^	Simulation PT; EV	PT can contaminate hands with bacteria. Recycled pulp has higher bacterial load *vs* virgin wood pulp. No difference between air contamination PT *vs* EV
Margas *et al*. (2013), UK	SCA Hygiene Products*	Simulation PT; JD	Settle plate and floor concentrations significantly higher with JD *vs* PT; No significant difference in airborne counts. JD use spread more bacteria to greater distances
Best *et al*. (2014), UK	European Tissue Symposium*	Simulation; Observational PT; WD; JD	PT contributed significantly less to the contamination (bacteria and paint) of the bathroom environment and to bystanders. PT *vs* WD mean counts at 1m distance were 7·8 and 0·7 CFU respectively
Best and Redway (2015), UK	European Tissue Symposium*	Observational; Intervention PT; CT; WD; JD	JD, WD and PT dispersed liquid from users’ hands up to 1·5, 0·75 and 0·5 m respectively
Jensen *et al*. (2015), US	USFDA^†^	Simulation PT; EV	Greater bacterial tracer reductions with PT *vs* EV (statistically significant: 1·9 *vs* 1·4 log10)
Kimmitt and Redway (2016), UK	European Tissue Symposium*; University of Westminster research reserve^†^	Simulation PT; WD; JD	JD air contamination and dispersal distances were significantly greater than WD and PT
Wilcox *et al*. (2017), UK	European Tissue Symposium*	Observational pilot PT; JD	Bacterial burden in bathrooms generally greater with JD *vs* PT. Differences statistically significant on dispenser surfaces and floors but not in air samples
Best *et al*. (2018), UK; France; Italy	European Tissue Symposium*	Observational PT; JD; CL	Significant differences in bacterial levels with PT generally lower than JD use over multiple scenarios (PT dispensers *vs* JD surfaces; floors)
Huesca‐Espitia *et al*. (2018), US	Consejo Nacional de Ciencia y Tecnología^†^	Observational WD	Plates exposed to hand dryer air averaged 18–60 colonies/plate, compared to plates exposed to bathroom air with dryers off, which averaged ≤1 colony. HEPA filters reduced bacterial dispersal ~fourfold
Pitt *et al*. (2018), UK	No financial support	Observational PT; WD; JD	PT decreased, WD increased and JD made no significant change to number of microbial flora on hands. JD and WD dispersed more microorganisms than PT
Mutters and Warnes (2019), Germany	Dyson*	Intervention JD; PT	Significantly fewer transient and resident bacteria after JD compared to PT. Increased number of opportunistic pathogens on PT dried hands

PT, paper towels; CT, cloth towel; JD, jet air dryer; WD, warm air dryer; EV, evaporation; CL, clothing.

Observational = field study; simulation = Lab study; intervention = pre‐post study design.

* Private funding.

^†^ Public funding.

Included articles either evaluated one hand‐drying method or compared the use of hand towels (paper or cloth) with ED (jet or warm air). Studies frequently utilized laboratory simulations targeting indigenous microbiomes of the environment and hands, or microbial tracers, to determine which hand‐drying method correlated with the least amount of hand, air or surface contamination. Two studies used nonmicrobial aerosol tracers (i.e. acid droplets or paint) to assess travel distances following specific drying behaviours and one study used a risk model approach to estimate probabilities of hand contamination.

A greater number of studies favoured the use of PT over air dryers based on comparisons of bacterial count trends on surfaces or in the air. Some studies found higher counts on PT dispensers. Even hands‐free towel dispensers were reportedly contaminated due to frequent contact with dispenser exit surfaces. Levels varied with malfunction frequency and user height (Harrison *et al*. 2003).

One study recommended a combined approach using towels followed by warm air‐drying to minimize bacterial transfer rates and maximize drying efficacy (Patrick *et al*. 1997). Given that wet hands transfer more bacteria between surfaces, improved efficacy of air dryers was an identified benefit in some studies. Rubbing with PT resulted in fewer bacterial counts likely due to the transfer of indigenous bacteria to the towel. No discussion was given to how this phenomenon relates to health. Little is known about the role of skin microbiota but authors suggested a beneficial function to these commensal organisms (Cogen *et al*. 2008).

Interpretation of the validity of study conclusions is complicated by variable experimental designs and fluctuating results between and within studies. Commonly referenced hand‐drying studies showed distinctly variable results. Some studies concluded that PT were more hygienic than dryers (Blackmore 1989; Ngeow *et al*. 1989a; Knights *et al*. 1993), while others found air dryers improved air quality (Ansari *et al*. 1991; Yamamoto *et al*. 2005). Several studies found no significant difference between the two (Matthews and Newsom 1987; Gustafson *et al*. 2000; Taylor *et al*. 2000).

A more recent study included a multi‐site, multi‐variable design and found that contamination levels in bathrooms with PT were generally lower than those with jet dryers (Best *et al*. 2018). However, many variables were identified that could have confounding effects and data trends were not always significant. Even for statistically significant scenario comparisons, questionable is the health risk associated with low contamination levels (i.e. 1 *vs* 2 MSSA organisms; 7 *vs* 21 MRSA organisms) when pathogenic bacteria infectious doses may be 5 or more orders of magnitude higher (Rose and Haas 1999). To our knowledge, high levels of baseline pathogenic bacteria concentrations have not been reported in real‐world bathroom or hand‐drying comparisons.

Best *et al*. (2018) found significant differences with colony counts of *Enterobacter* and enterococci where zero colonies compared to 1–2 log concentrations were reported in bathrooms using towels *vs* dryers, respectively. Both *Enterobacter* and enterococci are widespread in the environment and used to evaluate water or surface treatment interventions. While each can cause infections, including urinary tract infections, they are more commonly considered harmless commensals in the human gut and do not indicate a direct human health risk when detected in the environment.

For data to be meaningful in terms of a potential health risk associated with drying methods or environmental contamination levels, more research is needed to evaluate hazard prevalence and virulence; exposure routes, frequency and duration; dose–response potentials following exposure; and an overall characterization of risk. Epidemiological studies, particularly those assessing low levels of disease incidence, often require a large sample for an appropriately powered study. Such studies can be cost prohibitive and lack the sensitivity needed to assess low‐dose, low‐risk events. Previously developed tools in quantitative microbial risk assessment (QMRA) have been used to evaluate health outcomes related to pathogen exposures from environmental surfaces (Beamer *et al*. 2015; Wilson *et al*. 2018; Canales *et al*. 2019). Such computational tools could be applied to characterize surface contamination levels associated with various handwashing and hand‐drying methods, assess exposure potentials and forecast risk probabilities. Despite inferring health risks, none of the papers published to date considered a quantitative exposure or risk assessment approach to relate environmental contamination levels with human health outcomes. Furthermore, information is lacking relating seeded tracer concentrations with real‐world pathogen exposure levels. Extending risk estimates from tracer bacteria concentrations overestimates risk, as most of these starting concentrations do not represent expected pathogen levels (Ryan *et al*. 2014; Wilson *et al*. 2019). Consideration of pathogen transmission and environmental exposure potentials is needed for adequate assessment of exposures risks. For example, contamination of toilet seats and floors may not be a high‐risk event if they are not part of the exposure route leading to infection. In comparison, potentially lower contamination on a towel dispenser handle in direct and frequent contact with the hand may result in a greater risk of exposure to the mouth or other orifices. Exposure and activity data are critical to linking environmental contamination with risk and should be considered, along with handwashing efficacy, as a precursor to drying efficacy.

### Strengths and weaknesses

Of the 23 studies evaluated, all had a varying degree of scientific rigor, strengths and weaknesses (Table 3). These strengths and weaknesses were considered when assessing the validity of study conclusions. Performing a statistical analysis on the data generated from the experiment was considered a positive study attribute. Of the 23 scientifically reviewed studies, 22 were experimentally based (non‐computer simulations) and 17 of those conducted a statistical analysis. Studies that employed a statistical analysis plan sometimes favoured PT (PT) or electric (jet and warm air) dryers (ED) (Taylor *et al*. 2000; Yamamoto *et al*. 2005), or in some cases neither (Matthews and Newsom 1987; Gustafson *et al*. 2000; Yamamoto *et al*. 2005; Margas *et al*. 2013; Kimmitt and Redway 2016; Wilcox *et al*. 2017). In cases where statistics were not utilized, conclusions were the result of trend analysis, which can be misleading, especially in cases of large data variability or low contamination levels with a questionable relationship to significant health risks.

**Table 3:** Strengths and weaknesses of included hand drying articles

Citation	Strengths	Weaknesses
Matthews and Newsom (1987)	Statistical analysis	Experimental conditions lacked real‐world variable effects
Blackmore (1989)	Statistical analysis; large sample size (*n* > 110)	Unrealistic wash and drying conditions; used just HPCs; Low concentrations
Ngeow *et al*. (1989)	Realistic seed concentrations	No statistical comparisons; low limit of detection values; no aerosol sampling.
Ansari *et al*. (1991)	Tested virus and bacteria; statistical analysis; single volunteer for all methods	Unrealistic handwashing conditions; small sample size
Hanna *et al*. (1996)	Optimized laboratory recovery methods	No statistical analysis; unrealistic conditions; large seed concentrations
Patrick *et al*. (1997)	Small sample size (*n* = 7) but large number of replicates (*n* ≥ 36)	Towels autoclaved prior to use; no statistical analysis
Gustafson *et al*. (2000)	Advanced statistical analysis; large sample size (*n* = 99); Randomized study design; high seed concentrations but implemented a realistic handwashing scenario	No evaluation of real‐world microbial contaminants
Taylor *et al*. (2000)	Statistical analysis used with a 5% confidence level for significance	Dry times not consistent; volunteers instructed to dry hands until they felt dry
Montville *et al*. (2002)	Model simulations (*n* = 1000) validated with experimental data (*n* = 30)	Data compiled from highly diverse studies with data quality uncertainties
Harrison *et al*. (2003)	Performed statistical analysis; large number of replicates	Did not compare dryers and towels
Yamamoto *et al*. (2005)	Statistical analysis	Starting counts sometimes low‐ less than 10; data varied across different hand locations; inconsistent results; complicated indigenous flora parameter‐ no evidence of threat
Snelling *et al*. (2011)	Statistical analysis; used real‐world scenario to contaminate hands (handling raw meat followed by handwashing)	Data highly variable; comparisons not always significant; failed to test for pathogens
Gendron *et al*. (2012)	Considered background PT contamination impacts	No statistical comparisons; no risk evaluations
Margas *et al*. (2013)	Statistical analysis; controlled environmental conditions; Large number of volunteers (*n* = 100)	Data highly variable among participants
Best *et al*. (2014)	Statistical analysis; multiple tracers used	High inoculum; paint not representative of real‐world conditions
Best and Redway (2015)	Utilized both chemical and microbial tracers	No statistical analysis; high microbial burden may exaggerate transmission potentials; difficult to control reproducibility of volunteer behaviours; assumed handwashing is suboptimal; Risk not evaluated
Jensen *et al*. (2015)	Statistical analysis; pathogen surrogate tracers	Lack comparison to WD or JD method; Large data variability; Information lacking on data distributions; Exaggerated bacterial concentrations
Kimmitt and Redway (2016)	Statistical analysis; used virus surrogates	High seed concentrations; Lack comparison of WD *vs* PT
Wilcox *et al*. (2017)	Statistical analysis; targeted HPC and faecal indicator bacteria	Small pilot study (26 sampling days, single site, 2 washrooms, up to 5 swabs per locale); Information lacking on data distributions
Best *et al*. (2018)	Statistical analysis; large sample size (*n* = 120 sampling sessions); Multi‐site, multi‐scenario analysis; targeted faecal indicators and pathogenic bacteria	Highly variable site conditions; Low concentration differentials; Upper detection limit of 300 CFU; Information lacking on data distributions
Huesca‐Espitia *et al*. (2018)	Statistical analysis; multiple test organisms; VARIOUS air filtration methods	Low concentrations; data details not shown
Pitt *et al*. (2018)	Statistical analysis; utilized realistic handwashing and drying scenario; identified sampled organisms	Detailed data not provided; inconsistent drying times; methodology difficult to replicate and may not provide accurate counts
Mutters and Warnes (2019)	Statistical analysis; large number of volunteers (*n* = 80); bacterial tracer and resident flora; controlled and consistent environmental conditions; incorporated handwashing and evaluated drying and no drying variables	No environment sampling; drying methods may not be realistic

In addition to a lack of statistical analysis, results often lacked information on data distributions and summary statistics. Detailed summary statistics are helpful in analysing study power and data outliers that could be contributing to erroneous conclusions. Without this information, it is difficult to verify statistical methods, compare results across studies or utilize data to inform a QMRA model.

Sample size was also assessed and ranged from a few participants to >100. Larger sample sizes often led to application of statistical testing and thus were considered a stronger attribute compared to studies with smaller sample sizes. A large sample size is especially imperative in studies with large data variability in subjects, test organisms or environmental characteristics. Subject participation varied throughout the studies. In some cases, participants were required to dry their hands using only one method, while other studies reduced uncertainty between subjects by evaluating the same volunteers across multiple methods. In cases where participants utilized multiple drying methods, more weight was given to studies that randomized the drying method order to reduce participant bias.

Types of target organisms were also considered either a study strength or weakness. Targeting multiple organisms (i.e. HPC and faecal coliforms), instead of single tracer types, was considered a strength. Targeting one organism can result in bias, especially for naturally occurring skin bacteria that could be over/under‐represented based on handwashing practice and efficacy. The use of microbial tracers was preferred over indigenous skin microbes as a more consistent representation of baseline concentration. A few studies looked at chemical tracers, such as paint, to evaluate the spread of contamination. While providing useful information on the direct contamination around dryers, these methods do not necessarily correlate with microbial transfer, pathogen survival, exposure potentials or health outcomes.

Tracer application to hands also affected data variability. Many studies relied on natural contamination of the hands and environments. Other studies inoculated hands at either exaggerated concentrations or levels indicative of real‐world conditions. The hand/microbe interface is complex and impacted by friction, soap/hand sanitizer use, human cell–microbe interaction or adhesion factors, and the presence of organic matter. Some studies seeded gloved hands to minimize cell–microbe interactions. Such unrealistic conditions were considered a study weakness as they create uncertainty relative to the effect of surface interactions on pathogen transfer.

Study location was another source of uncertainty, ranging from biosafety hoods to laboratory washrooms to fully functioning bathrooms. Studies completed in biosafety hoods and laboratory washrooms did not take into consideration real‐world effects of human traffic and behaviours. This should be considered when evaluating conclusions or comparing results across studies. Studies utilizing fully functioning bathrooms often did not provide a detailed description of the bathroom layout, which makes it difficult to compare results to other studies given room influences on microbe dispersion due to airflow, room size, and placement of walls and hand‐drying dispensers.

Included studies ranged from the 1980s to present day. In this ~40‐year time span, air dryer and PT dispenser technology evolved. For example, automated towel dispensers reduced hand contact with potentially contaminated handle surfaces but did not eliminate this exposure route completely due to frequent malfunctions (Harrison *et al*. 2003). Jet dryer technology reduced dry times to approximately 10 s compared to 30 s with warm air dryers but was associated with increased concentrations and dispersion rates of indigenous microbes and tracers in several studies.

All studies agreed on the importance of effective hand drying but consumer preference ranged across methods and consideration of hygiene perceptions, speed and drying efficacy with 63% of 2008 survey respondents preferring PT to warm air dryers (28%) or cloth towels (10%) (Todd *et al*. 2010). Given that the survey, and much of the historical research on hand‐drying hygiene and efficacy, was completed prior to some of the later hand‐drying innovations (i.e. automated towel dispensers, jet dryers or HEPA‐filtered dryer air) perceptions and data analysis today may be different.

Variable applications and technologies complicated comparisons between studies in our analysis. In some studies, detailed information regarding dryer or towel dispenser technology was included, while others only list general descriptions. Few studies evaluated newer drying innovations such as hands‐free PT dispensers or HEPA filtered air dryers on tracer or microbial concentrations, which could have an impact on the overall conclusions of technology comparisons.

### Data summary of major findings and study rigor

Table 4 is a semi‐quantitative characterization of major study findings and some highly referenced grey literature, sorted relative to descending rigor. Light grey boxes indicate qualitative and quantitative study strengths (i.e. large sample size, statistical significance, controlled variables, advanced statistics) and were assigned a value of two. Minus signs (dark grey boxes) indicate weaknesses relative to variable consistency, methodology, realistic conditions, data quality and other parameters (i.e. result inconsistencies) and were assigned a value of zero. White boxes were assigned a value of one relative to neutral assessments of defined qualitative and quantitative parameters (i.e. determined to be neither a strength nor weakness). An evaluation of the scenario indicates whether study conclusions generally favoured ED, PT or both equally. A scenario category of ‘N/A’ was assigned for studies that did not compare hand dryers to PT. The studies with the highest relative rigor scores (11 and 10) favoured ED or neither method; however, studies with a relative score equal to or above a six (*n* = 11) favoured PT (*n* = 4), ED (*n* = 2), mixed results (*n* = 3) or did not compare multiple methods (*n* = 2).

**Table 4:** Quantitative study rigor assessment of hand drying studies

Citation*	Favoured scenario	Mixed results^†^	Sample size	Variable consistency	Methodology	Realistic conditions	Data quality	Other	Funding	Relative score^§^
Gustafson *et al.* (2000)	ED> PT PT = ED	X	+ 2	+ 2	+ 2	Neutral 1	+ 2	+ 2	Private	11
Mutters and Warnes (2019)	ED> PT		+ 2	+ 2	Neutral 1	Neutral 1	+ 2	+ 2	Private	10
Ansari *et al.* (1991)	ED> PT		− 0	+ 2	+ 2	− 0	+ 2	Neutral 1	N/A^‡^	7
Wilcox *et al.* (2017)	PT> ED PT = ED	X	− 0	Neutral 1	+ 2	+ 2	Neutral 1	Neutral 1	Private	7
Huesca‐Espitia *et al.* (2018)	N/A		Neutral 1	Neutral 1	+ 2	Neutral 1	Neutral 1	Neutral 1	Public	7
Best *et al.* (2014)	PT> ED		Neutral 1	Neutral 1	+ 2	− 0	+ 2	Neutral 1	Private	7
Montville *et al.* (2002)	PT> ED		+ 2	Neutral 1	+ 2	Neutral 1	− 0	Neutral 1	Private	7
Pitt *et al.* (2018)	PT> ED		Neutral 1	Neutral 1	Neutral 1	+ 2	Neutral 1	− 0	None	6
Harrison *et al.* (2003)	N/A		+ 2	− 0	− 0	Neutral 1	+ 2	Neutral 1	Private	6
Blackmore (1989)	PT> ED		+ 2	Neutral 1	− 0	− 0	+ 2	Neutral 1	Private	6
Matthews and Newsom (1987)	ED> PT PT = ED	X	Neutral 1	Neutral 1	Neutral 1	− 0	+ 2	Neutral 1	Public	6
Gendron *et al.* (2012)	N/A		− 0	Neutral 1	+ 2	− 0	Neutral 1	Neutral 1	Public	5
Patrick *et al.* (1997)	N/A		+ 2	Neutral 1	− 0	Neutral 1	− 0	Neutral 1	Private	5
Snelling *et al.* (2011)	PT> ED PT = ED	X	Neutral 1	Neutral 1	− 0	Neutral 1	Neutral 1	Neutral 1	Private	5
Margas *et al.* (2013)	PT> ED ED> PT PT = ED	X	+ 2	− 0	Neutral 1	− 0	Neutral 1	Neutral 1	Private	5
Best *et al.* (2018)	PT> ED PT = ED	X	Neutral 1	Neutral 1	Neutral 1	Neutral 1	Neutral 1	− 0	Private	5
Hanna *et al.* (1996)	PT> ED		Neutral 1	Neutral 1	Neutral 1	− 0	− 0	+ 2	Private	5
Taylor *et al.* (2000)	ED> PT PT = ED	X	− 0	− 0	Neutral 1	Neutral 1	Neutral 1	Neutral 1	Private	4
Ngeow *et al.* (1989)	PT> ED		− 0	− 0	Neutral 1	+ 2	− 0	Neutral 1	Public	4
Best and Redway (2015)	PT> ED		Neutral 1	− 0	+ 2	− 0	− 0	Neutral 1	Private	4
Jensen *et al.* (2015)	N/A		Neutral 1	− 0	Neutral 1	− 0	Neutral 1	Neutral 1	Public	4
Kimmitt and Redway (2016)	PT> ED PT = ED	X	− 0	Neutral 1	Neutral 1	− 0	Neutral 1	− 0	Private	3
Yamamoto *et al.* (2005)	PT> ED ED> PT	X	− 0	− 0	− 0	Neutral 1	Neutral 1	− 0	Public	2

ED, electric dryer; PT, paper towels; N/A, comparisons between PT and ED not performed; neutral = one; positive (+) = two.

All boxes with a score of 2 should have a lighter gray shade.

All boxes with a score of zero should have a darker gray shade.

All boxes with a score of 1 should have no shading.

* Listed in descending order according to rigor score.

^†^ Method favoured varied over changing scenarios.

^‡^ Funding source not reported.

^§^ Study rigor scoring key: negative (−) = zero.

### Electric dryers favoured

Of the 23 papers of determined relevance, 7 had results supporting the use of ED over PT. Reasons for this conclusion ranged from observations of less bacterial counts in the environment or on external device surfaces (Matthews and Newsom 1987; Ansari *et al*. 1991; Gustafson *et al*. 2000; Taylor *et al*. 2000; Yamamoto *et al*. 2005; Mutters and Warnes 2019) or less concentration of liquid droplets on surfaces near towel or dryer devices (Margas *et al*. 2013). However, most of the studies favouring ED over PT showed mixed results in terms of statistically significant data depending on which variables were evaluated. Often, within the same study, conditions favoured ED under certain scenarios and PT in others (Margas *et al*. 2013). Others evaluated results statistically but found no significant difference between hand‐drying methods (Gustafson *et al*. 2000; Taylor *et al*. 2000; Margas *et al*. 2013). Four studies favouring ED were funded in part by private sources (Gustafson *et al*. 2000; Taylor *et al*. 2000; Margas *et al*. 2013; Mutters and Warnes 2019), the remaining three were funded by what appeared to be independent or public sources (Matthews and Newsom 1987; Ansari *et al*. 1991; Yamamoto *et al*. 2005).

### Paper towels favoured

Thirteen papers generally favoured PT use over ED and seven of these studies showed statistically significant differences (Blackmore 1989; Yamamoto *et al*. 2005; Snelling *et al*. 2011; Best *et al*. 2014, 2018; Kimmitt and Redway 2016; Pitt *et al*. 2018). Five of the 13 studies were funded by The European Tissue Symposium (Best *et al*. 2014, 2018; Best and Redway 2015; Kimmitt and Redway 2016; Wilcox *et al*. 2017) and 4 were conducted by the same research collaborators. Five additional studies that generally supported PT use over ED were sponsored by other private funding sources (Blackmore 1989; Hanna *et al*. 1996; Montville *et al*. 2002; Snelling *et al*. 2011; Margas *et al*. 2013). Two others were funded what appeared to be independent or public sources (Ngeow *et al*. 1989b; Yamamoto *et al*. 2005). One study reportedly received no funding (Pitt *et al*. 2018).

### Paper towels equal to electric dryers

Eight papers found, at least in part, that PT were equally favoured as ED (Matthews and Newsom 1987; Gustafson *et al*. 2000; Taylor *et al*. 2000; Snelling *et al*. 2011; Margas *et al*. 2013; Kimmitt and Redway 2016; Wilcox *et al*. 2017; Best *et al*. 2018). At least three of these studies conducted statistical analysis but found no difference between variables regardless of drying method used. Specifically, Taylor *et al*. (2000) found no significant difference in microbial aerosols created from ED compared to PT. Others also found that PT and ED were not significantly different in terms of contamination potentials from microbial aerosols under a variety of test conditions (Gustafson *et al*. 2000; Margas *et al*. 2013; Kimmitt and Redway 2016; Wilcox *et al*. 2017; Best *et al*. 2018).

### Miscellaneous study results

Five papers failed to evaluate PT and ED in the same study (Patrick *et al*. 1997; Harrison *et al*. 2003; Gendron *et al*. 2012; Jensen *et al*. 2015; Huesca‐Espitia *et al*. 2018). These studies provided useful background information relative to other variables of potential interest. For example, Gendron *et al*. (2012) focused on the potential for PT to add bacteria to the environment. Sources include indigenous organisms found on the product post‐manufacturing but pre‐use, particularly with recycled pulp products, and further contamination potentials during transport to dispensers (Gendron *et al*. 2012).

### Impact of study rigor

A common observation in our analysis is that most of the studies, regardless of conclusion, lacked sufficient rigor to form defensible conclusions. Some utilized small sample sizes (Kimmitt and Redway 2016; Wilcox *et al*. 2017) or unrealistic conditions, such as evaluating how paint spreads (Best *et al*. 2014), which may have no relationship to bacterial transmission. Others did not consider either handwashing or soap use before drying (Snelling *et al*. 2011; Best and Redway 2015; Jensen *et al*. 2015), which may exaggerate results given that washing is an expected drying precursor for contaminated hands. Furthermore, highly variable results within and between scenarios was a common effect (Taylor *et al*. 2000; Yamamoto *et al*. 2005; Margas *et al*. 2013; Best and Redway 2015; Jensen *et al*. 2015). Several authors made conclusions on the safety of different hand‐drying methods that were not supported by the data (Ngeow *et al*. 1989a; Gould 1994; Kimmitt and Redway 2016). Regardless of scenario, most studies lacked objective statistical significance testing or failed to define test criteria (Ngeow *et al*. 1989a; Best *et al*. 2014; Kimmitt and Redway 2016).

### Grey literature summary

In 2018, there was a resurgence of news interest on the topic of PT *vs* hand dryers due to newer published research (Best *et al*. 2014, 2018; Best and Redway 2015; Kimmitt and Redway 2016; Huesca‐Espitia *et al*. 2018). In addition, several grey literature reports went ‘viral’ on the Internet, such as the photo of a Petri dish placed in a jet air dryer as part of a small class experiment (Astor 2018). Grey literature includes materials (i.e. research, white papers, media and industry reports, government documents, surveys, theses and dissertations, data summaries, etc.) produced by individuals or organizations outside the traditional peer review process. While the classification of information as grey literature does not necessarily mean the data is not scientifically valid, there may be less oversight and review of the content. Here we included a review of the grey literature using the top Google results when searching ‘PT air dryers’. We also included sources disqualified during the scoping review, such as papers that did not contain original research or were not peer‐reviewed (Gould 1994; Lee 1994; Huang *et al*. 2012; Haynes 2014).

To extract information from the grey literature, we initially reviewed known popular sources, including Dr. Oz, MythBusters, the New York Times, Washington Post, Snopes.com and Oprah.com. Most of these reports feature headlines suggesting there may be a more hygienic way to dry hands (Table 5). The majority of these outlets favoured PT based on cited research from peer‐reviewed or other grey literature sources but often stated that data was inconclusive relative to safety concerns. Some media outlets performed their own small‐scale experiments, or simply cited personal preference.

**Table 5:** Media headlines related to hand‐drying methods

Article title	Reference
‘Which is better in a commercial restroom? Hand dryers or paper towels?’	Restroom Directn.d. (‘Which is better in a commercial restroom? Hand dryers or paper towels?’)
‘Paper towels prove most hygienic in restroom study’	Convenience Store Decisions 2009 (‘Paper Towels Prove Most Hygienic in Restroom Study’ 2009)
‘Down and dirty, earthquake survival’	MythBusters 2013 (‘MythBusters Episode 199: Down and Dirty, Earthquake Survival’ 2013)
‘Best way to wash hands in public’	Oprah.com 2015 (‘Best Way To Wash Hands in Public’ 2015)
‘The hygienic way to dry your hands’	Dr. Oz 2017 (‘The Hygienic Way to Dry Your Hands’ 2017)
‘Hand dryers can blow fecal bacteria onto your hands, a study found—and the researchers are now switching to paper towels’	Business Insider 2018 (Brueck 2018)
‘There’s poop in bathroom hand dryers’	Care2 2018 (Donsky 2018)
‘FACT CHECK: Do hot air hand dryers in restrooms spread disease?’	Snopes 2018 (‘FACT CHECK: Do Hot Air Hand Dryers in Restrooms Spread Disease?’ 2018)
‘Hand dryers spread bacteria so dramatically that scientists think they're a public health threat’	IFLScience 2018 (Kovner 2018)
‘How dirty are hand dryers?’	Dr. Oz 2018 (‘How Dirty Are Hand Dryers?’ 2018)

Some of the data in the grey literature misconstrued research results. For example, multiple online articles about the Huesca‐Espitia *et al*. (2018) study state that PT are a better drying option and that dryers blow faecal matter on hands, which is not completely reflective of the authors’ conclusions. The Huesca‐Espitia *et al*. (2018) study did not evaluate PT or run any comparative tests between dryers and towels. The paper does voice concern over dryers potentially blowing pathogens around bathroom environments but also reports very low counts of microbes and was favourable towards warm air dryers with HEPA filters that reduced counts by fourfold.

Few of the grey literature reports provide citations or links to publications referenced. Many of the findings were overgeneralized and included unsubstantiated claims, Internet rumours and inaccurate information reported as fact. According to Snopes.com, a popular fact‐checking website, an email was circulated in 2009 claiming schools and universities were required to replace all hand dryers with towel dispensers in response to the H1N1 outbreak ('FACT CHECK: Do Hot Air Hand Dryers in Restrooms Spread Disease?' 2018). In its analysis, Snopes rates the claim as ‘unproven’.

One of the concerns with the grey literature reviewed in our study is the potential bias in the reporting. Some of the top‐ranking articles in our Google search were white papers authored or sponsored by paper companies (Vitalin.d.; Lee 1994). Additionally, media reports frequently used sensationalized headlines. While such headlines may increase traffic, they sometimes overgeneralize or exaggerate study results. Consumers may only read headlines which can influence public opinion towards biased or erroneous conclusions (Ecker *et al*. 2014). Ecker *et al*. also found that readers are more likely to recall details of the article relative to expectations set by inferences in the headline and not by what the article actually says.

## Conclusion

The objective of our study was to provide a scoping review of the current scientific peer‐reviewed publications comparing the use of PT *vs* ED and their impact on environmental and human health using standardized criteria. Our focus was to categorize the papers relative to scientific rigor in the study design and compare statistically significant results across defined categories of hand‐drying methods. We also examined statistically significant results relative to paper conclusions and the impact of these studies on public perception (as determined by media posts and other grey literature). Study categories were scored quantitatively as positive/+, neutral or negative/−, using a scoring system of 2, 1 and 0 respectively. While it is important to review each study holistically, providing a study rigor score to each reviewed paper adds context to our assessment, which should be read in full for a broader perspective.

The most prevalent conclusion from our scoping review is that the breadth of scientific data does not support a uniform conclusion as to which drying method is safer or more hygienic. Some studies showed a statistically significant reduction of contaminants within certain scenarios but not all‐ whether studies involved large sample sizes or were small pilot studies, the differences between drying methods were not consistently clear. Many studies reported no statistical significance and used either low concentrations representing low‐risk scenarios or high concentrations that are not representative of pathogen levels on hands or bathroom surfaces.

Overall, the large discrepancy in results is likely due to the wide range of experimental conditions implemented. Variables include: baseline concentrations, target organisms, population and sample sizes, drying times, number of towels used, dispenser designs, rubbing behaviours, microbial assay methods, Hawthorne effects, behavioural reproducibility, handwashing product types and efficacy, skin integrity, residual moisture levels, ambient air movement and more.

Methodological differences compounded these uncertainties. Mixed results favouring one drying method over another between, and even within studies, was common. None of the studies in the published literature related environmental contamination differentials of any drying method with health outcomes. However, several studies and highly referenced reviews and reports have made recommendations as to which methods to use, or avoid, based on qualitative safety or health claims, examples include:

- ‘There seems to be no particular microbiological danger to use of hot air hand dryers’. (Matthews and Newsom 1987)
- ‘Hot air dryers are generally not recommended for use in health care settings because such dryers are relatively slow and noisy and their hygiene performance is questionable’ (Blackmore 1989)
- ‘…associated infection risks…make [air dryers] unsuitable for use in critical patient care areas’ (Ngeow *et al*. 1989a).
- ‘…the use of paper towels is safer than hot air hand‐dryers in busy wards’ (Gould 1994).
- ‘Risk reduction strategies should aim to achieve a level of risk as low as practically possible’ (Harrison *et al*. 2003).
- ‘The health and safety aspects of jet air dryers where hygiene is paramount should still be carefully examined by the scientific community’ (Redway and Fawdar 2008).
- ‘…a diverse community of culturable bacteria contaminates unused paper towels…some of these bacterial strains may be toxin producers…This may be significant for some clinical and industrial settings as well as for immunocompromised individuals…’ (Gendron *et al*. 2012).
- ‘From a hygiene standpoint, paper towels are superior to air dryers’ (Huang *et al*. 2012).
- ‘These results suggest that air dryers may be unsuitable for use in healthcare settings as they may facilitate microbial cross‐contamination via airborne dissemination to the environment or bathroom visitors’ (Best *et al*. 2014).
- ‘…in locations where hygiene and cross‐infection considerations are paramount, such as healthcare settings and the food industry, the choice of hand‐drying method should be considered carefully’ (Kimmitt and Redway 2016).
- ‘Electric hand dryers are not suited to clinical settings… Infection control building guidance needs to be amended and strengthened’ (Best *et al*. 2018).

Some studies stated concerns relative to the use of air dryers in healthcare settings. It is important to note, however, that the most effective, promoted and commonly practiced hand hygiene method in hospitals and other healthcare settings involves the use of an alcohol‐based hand rub (Vermeil *et al*. 2019). In healthcare settings, alcohol‐based hand rubs require less time, are more effective than washing with soap and water, are more accessible than sinks and naturally exclude the use of hand‐drying protocols (Boyce and Pittet 2002; World Health Organization 2009).

Increased bacterial counts in the environment do not necessarily indicate increased health risks. To date, no study has shown a correlation between hand‐drying methods and health outcomes using empirically derived or QMRA model simulations. Thus, conclusions as to the safety of PT *vs* air dryer use relative to health outcomes are unsubstantiated and premature. Consideration of infective doses, transmission pathways, handwashing and drying efficacy, transfer rates and other exposure variables is critical before a recommendation can be made based on health outcomes.

Not all surfaces are equal contributors to exposure pathways. While floors may be more contaminated in air dryer bathrooms, their relevance in terms of an exposure or risk scenario has not been evaluated. The most direct exposure potential is from contaminated hands and thus drying methods resulting in fewer microbes on the hand are likely to be associated with lower risk potentials. Therefore, contaminated dryer or PT dispenser buttons may represent the highest risk in practice. Conversely, new technologies such as automated dryer and towel dispenser designs may largely eliminate this transmission route.

Our scoping review identified broad analysis of the research question: ‘Are hand dryers more hygienic than PT?’ but with collectively inconclusive results. Studies were conducted by a variety of private and independent research groups spanning at least nine countries. Fifteen of the 23 studies were funded by private, or a combination of private and public, sponsors. Although independent funding is preferred, federal or state funding agencies are unlikely to sponsor such special interest projects‐ particularly when current research does not indicate a significant health concern.

The second question we sought to answer is ‘Are PT safer than hand dryers relative to human infection risks?’ We found no data to support any human health claims relative to hand dryers *vs* PT use. Inconclusive results in the current body of research and a lack of data to support health claims represent data gaps preventing the advancement of policy or practice recommendations relative to hand‐drying protocols. Of notable importance is the need to evaluate risks from hand‐drying activities in consideration of handwashing scenarios, given that the greatest uncertainty in hand contamination is associated with the handwashing method, and not the drying method.

## Future study recommendations

Future studies are needed to examine the relationship between contamination that occurs due to hand‐drying methods and human health outcomes. This can be accomplished by utilizing a real‐world scenario while still controlling for certain variables. Ideally, the study should be done in multiple functioning bathrooms that have different characteristics, such as single stall and multiple stall bathrooms. A microbial tracer should be applied to participants’ ungloved hands at a concentration that would be representative of what is expected on hands after a handwashing event. The hands of the participants and surfaces that participants would encounter after drying their hands should be targeted for sampling as well as air. Samples should be collected after single and multiple drying events to see the effects over a variety of usage conditions. Results from the study can then be used to inform mathematical models to evaluate human health outcomes expected for each type of drying event. Sensitivity analysis should be performed to rank which exposure variables drive risk outcomes.

In summary, needed are long‐term observational studies and/or health impact related evaluations/simulations. Gaps in the research include:

- Evaluation of contamination potentials with current technologies and standardized methodology.
- Consistent variable assessment representing real‐world exposure potentials (i.e. consideration of baseline contamination values, handwashing and drying efficacy, pathogen dose response, and correlation between indigenous microbiome or tracer concentrations to predicted pathogen levels).
- An exposure assessment and sensitivity analysis considering surfaces of increased probability to impact exposure.
- A risk assessment relating relevant exposure scenarios to health outcomes.
- A relative risk assessment comparing different contamination levels from other bathroom sources (i.e. toilet aerosols or human behaviours) and organism infectivity with acceptable risk targets.

Although numerous studies have been published evaluating the ‘best’ method for hand drying, ‘best’ has been defined in a variety of ways relative to bacterial removal efficacy, environmental contamination potentials, ecological or cost benefits, noise and more. No study to date has examined the ‘best’ drying method relative to health outcomes.

As reported repeatedly throughout this study, the wide range of data variability makes it difficult to draw finite conclusions about any particular hand‐drying method. We agree with the conclusions of Wilcox *et al*. (2017) who state that studies to determine the risks associated with hand‐drying methods have not been conducted but are warranted to determine whether adverse infection consequences occur.

## Funding support

This study was supported, in part, by an unrestricted grant from Excel Dryer, Inc. The funding source played no role in study design, data acquisition, analysis or decision to report these data.

## Conflicts of Interest

K.A. Reynolds has received research funds from Excel Dryer, Inc.

## References

[jam14796-bib-0001] Ansari, S.A. , Springthorpe, V.S. , Sattar, S.A. , Tostowaryk, W. and Wells, G.A. (1991) Comparison of cloth, paper, and warm air drying in eliminating viruses and bacteria from washed hands. Am J Infect Control 19, 243–249. 1661567 10.1016/s0196-6553(05)80256-1

[jam14796-bib-0002] Arksey, H. and O’Malley, L. (2005) Scoping studies: towards a methodological framework. Int J Soc Res Methodol 8, 19–32.

[jam14796-bib-0003] Astor, M. (2018) Are Hand Dryers Actually Full of Bacteria? A Viral Photo Doesn’t Tell the Whole Story. New York Times.

[jam14796-bib-0004] Beamer, P.I. , Plotkin, K.R. , Gerba, C.P. , Sifuentes, L.Y. , Koenig, D.W. and Reynolds, K.A. (2015) Modeling of human viruses on hands and risk of infection in an office workplace using micro‐activity data. J Occup Environ Hyg 12, 266–275. 25436665 10.1080/15459624.2014.974808 PMC4455933

[jam14796-bib-0005] Best, E. , Parnell, P. , Couturier, J. , Barbut, F. , Le Bozec, A. , Arnoldo, L. , Madia, A. , Brusaferro, S. *et al* (2018) Environmental contamination by bacteria in hospital washrooms according to hand‐drying method: a multi‐centre study. J Hosp Infect 100, 469–475. 30006281 10.1016/j.jhin.2018.07.002

[jam14796-bib-0006] Best, E.L. , Parnell, P. and Wilcox, M.H. (2014) Microbiological comparison of hand‐drying methods: the potential for contamination of the environment, user, and bystander. J Hosp Infect 88, 199–206. 25237036 10.1016/j.jhin.2014.08.002

[jam14796-bib-0007] Best, E.L. and Redway, K. (2015) Comparison of different hand‐drying methods: the potential for airborne microbe dispersal and contamination. J Hosp Infect 89, 215–217. 25586988 10.1016/j.jhin.2014.11.007

[jam14796-bib-0008] Best Way To Wash Hands in Public (2015) Oprah.com. Available at: http://www.oprah.com/omagazine/best‐way‐to‐wash‐hands‐in‐public.

[jam14796-bib-0009] Blackmore, M. (1989) A comparison of hand drying methods. Cater Health 1, 189–198.

[jam14796-bib-0010] Bolon, M.K. (2016) Hand hygiene: an update. Infect Dis Clin North Am 30, 591–607. 27515139 10.1016/j.idc.2016.04.007

[jam14796-bib-0011] Boyce, J.M. and Pittet, D. (2002) Guideline for hand hygiene in health‐care settings: recommendations of the healthcare infection control practices advisory committee and the HICPAC/SHEA/APIC/IDSA hand hygiene task force. Am J Infect Control 30, S1–S46. 12461507 10.1067/mic.2002.130391

[jam14796-bib-0012] Brueck, H. (2018) Hand Dryers Can Blow Fecal Bacteria onto Your Hands, A Study Found — and the Researchers are Now Switching to Paper Towels. Business Insider Available at: https://www.businessinsider.com/bathroom‐hand‐dryers‐or‐paper‐towels‐which‐carries‐more‐bacteria‐2018‐4.

[jam14796-bib-0013] Canales, R.A. , Reynolds, K.A. , Wilson, A.M. , Fankem, S.L.M. , Weir, M.H. , Rose, J.B. , Abd‐Elmaksoud, S. and Gerba, C.P. (2019) Modeling the role of fomites in a norovirus outbreak. J Occup Environ Hyg 16, 16–26. 30274562 10.1080/15459624.2018.1531131

[jam14796-bib-0014] Cogen, A.L. , Nizet, V. and Gallo, R.L. (2008) Skin microbiota: a source of disease or defence? Br J Dermatol 158, 442–455. 18275522 10.1111/j.1365-2133.2008.08437.x PMC2746716

[jam14796-bib-0015] Dean, S. (2018) Don’t Let The Internet Scare You With That Hand Dryer Petri Dish. Science Alert. Available at: https://www.sciencealert.com/hand‐dryer‐petri‐dish‐experiment‐facebook‐viral‐photo‐scary.

[jam14796-bib-0016] Donsky, A. (2018) There’s poop in bathroom hand dryers. Available at: https://www.care2.com/greenliving/theres‐poop‐in‐bathroom‐hand‐dryers.html.

[jam14796-bib-0017] Ecker, U.K.H. , Lewandowsky, S. , Chang, E.P. and Pillai, R. (2014) The effects of subtle misinformation in news headlines. J Exp Psychol Appl 20, 323–325. 25347407 10.1037/xap0000028

[jam14796-bib-0018] Snopes (2018) FACT Check: Do Hot Air Hand Dryers in Restrooms Spread Disease? Available at: https://www.snopes.com/fact‐check/blowing‐hard‐2/.

[jam14796-bib-0019] FDA (2017) FDA Food Code 2017. College Park, MD: US Department of Health and Human Services.

[jam14796-bib-0020] Gendron, L.M. , Trudel, L. , Moineau, S. and Duchaine, C. (2012) Evaluation of bacterial contaminants found on unused paper towels and possible postcontamination after handwashing: a pilot study. Am J Infect Control 40, e5–e9. 22177666 10.1016/j.ajic.2011.07.007

[jam14796-bib-0021] Gerba, C.P. , Lopez, G.U. and Ikner, L.A. (2016) Distribution of bacteria in dental offices and the impact of hydrogen peroxide disinfecting wipes. J Dent Hyg JDH 90. 29118156

[jam14796-bib-0022] Gould, D. (1994) The significance of hand‐drying in the prevention of infection. Nurs Times 33–35. 7800517

[jam14796-bib-0023] Grant, M.J. and Booth, A. (2009) A typology of reviews: an analysis of 14 review types and associated methodologies. Heal Inf Libr J 26, 91–108. 10.1111/j.1471-1842.2009.00848.x 19490148

[jam14796-bib-0024] Gustafson, D.R. , Vetter, E.A. , Larson, D.R. , Ilstrup, D.M. , Maker, M.D. , Thompson, R.L. and Cockerill, F.R. (2000) Effects of 4 hand‐drying methods for removing bacteria from washed hands: a randomized trial. Mayo Clin Proc 75, 705–708. 10907386 10.4065/75.7.705

[jam14796-bib-0025] Hafner, J. (2018) Ban jet air hand dryers? Scientists urge hospitals to avoid them, citing increased germs. USA Today.

[jam14796-bib-0026] Hanna, P.J. , Richardson, B.J. and Marshall, M. (1996) A comparison of the cleaning efficiency of three common hand drying methods. Appl Occup Environ Hyg 11, 37–43.

[jam14796-bib-0027] Harrison, W.A. , Griffith, C.J. , Ayers, T. and Michaels, B. (2003) Bacterial transfer and cross‐contamination potential associated with paper‐towel dispensing. Am J Infect Control 31, 387–391. 14639433 10.1067/mic.2003.81

[jam14796-bib-0028] Haynes, A. (2014) Paper towels versus hot air hand dryers — which is more hygienic? Pharmaceut J. Available at: https://www.pharmaceutical‐journal.com/opinion/blogs/paper‐towels‐versus‐hot‐air‐hand‐dryers‐which‐is‐more‐hygienic/20067412.blog.

[jam14796-bib-0030] Huang, C. , Ma, W. and Stack, S. (2012) The hygienic efficacy of different hand‐drying methods: a review of the evidence. Mayo Clin Proc 87, 791–798. 22656243 10.1016/j.mayocp.2012.02.019 PMC3538484

[jam14796-bib-0031] Huesca‐Espitia, L.C. , Aslanzadeh, J. , Feinn, R. , Joseph, G. , Murray, T.S. and Setlow, P. (2018) Deposition of bacteria and bacterial spores by bathroom hot‐air hand dryers. Appl Environ Microbiol 84, e00044‐18. 29439992 10.1128/AEM.00044-18 PMC5881072

[jam14796-bib-0032] Jensen, D.A. , Danyluk, M.D. , Harris, L.J. and Schaffner, D.W. (2015) Quantifying the effect of hand wash duration, soap use, ground beef debris, and drying methods on the removal of *Enterobacter aerogenes* on hands. J Food Prot 78, 685–690. 25836392 10.4315/0362-028X.JFP-14-245

[jam14796-bib-0033] Khalil, H. , Peters, M. , Godfrey, C.M. , McInerney, P. , Soares, C.B. and Parker, D. (2016) An evidence‐based approach to scoping reviews. Worldviews Evid‐Based Nurs 13, 118–123. 26821833 10.1111/wvn.12144

[jam14796-bib-0034] Kimmitt, P.T. and Redway, K.F. (2016) Evaluation of the potential for virus dispersal during hand drying: a comparison of three methods. J Appl Microbiol 120, 478–486. 26618932 10.1111/jam.13014

[jam14796-bib-0035] Knights, B. , Evans, C. , Barrass, S. and McHardy, B. (1993) Hand Drying: Assessment of Efficiency and Hygiene of Different Methods (pp. 1–16). London: Survey by the Applied Ecology Research Group at the University of Westminster.

[jam14796-bib-0036] Kovner, A. (2018) Hand Dryers Spread Bacteria So Dramatically That Scientists Think They’re A Public Health Threat. IFLScience Available at: https://www.iflscience.com/health‐and‐medicine/hand‐dryers‐spread‐bacteria‐so‐dramatically‐that‐scientists‐think‐theyre‐a‐public‐health‐threat/.

[jam14796-bib-0037] Lee, M. (1994) Paper and cloth towels found to be more hygienic than air dryers. Health Facil Manage 7, 114–116. 10136061

[jam14796-bib-0038] Levac, D. , Colquhoun, H. and O’Brien, K.K. (2010) Scoping studies: advancing the methodology. Implement Sci 5, 69. 20854677 10.1186/1748-5908-5-69 PMC2954944

[jam14796-bib-0039] Lopez, G.U. , Gerba, C.P. , Tamimi, A.H. , Kitajima, M. , Maxwell, S.L. and Rose, J.B. (2013) Transfer efficiency of bacteria and viruses from porous and nonporous fomites to fingers under different relative humidity conditions. Appl Environ Microbiol 79, 5728–5734. 23851098 10.1128/AEM.01030-13 PMC3754157

[jam14796-bib-0040] Margas, E. , Maguire, E. , Berland, C.R. , Welander, F. and Holah, J.T. (2013) Assessment of the environmental microbiological cross contamination following hand drying with paper hand towels or an air blade dryer. J Appl Microbiol 115, 572–582. 23683001 10.1111/jam.12248

[jam14796-bib-0041] Matthews, J.A. and Newsom, S.W.B. (1987) Hot air electric hand driers compared with paper towels for potential spread of airborne bacteria. J Hosp Infect 9, 85–88. 2880906 10.1016/0195-6701(87)90101-0

[jam14796-bib-0042] Montville, R. , Chen, Y. and Schaffner, D.W. (2002) Risk assessment of hand washing efficacy using literature and experimental data. Int J Food Microbiol 73, 305–313. 11934038 10.1016/s0168-1605(01)00666-3

[jam14796-bib-0043] Mutters, R. and Warnes, S.L. (2019) The method used to dry washed hands affects the number and type of transient and residential bacteria remaining on the skin. J Hosp Infect 101, 408–413. 30537524 10.1016/j.jhin.2018.12.005

[jam14796-bib-0044] MythBusters Episode 199: Down and Dirty, Earthquake Survival (2013) MythBusters Results. Available at: https://mythresults.com/down‐dirty‐earthquake‐survival.

[jam14796-bib-0045] Ngeow, Y.F. , Ong, H.W. and Tan, P. (1989a) Dispersal of bacteria by an electric air hand dryer. Malays J Pathol 11, 53–56. 2698982

[jam14796-bib-0046] Paper Towels Prove Most Hygienic in Restroom Study (2009) Convenience Store Decisions. Available at: https://cstoredecisions.com/2009/06/08/paper‐towels‐prove‐most‐hygienic‐in‐restroom‐study/.

[jam14796-bib-0047] Patrick, D.R. , Findon, G. and Miller, T.E. (1997) Residual moisture determines the level of touch‐contact‐associated bacterial transfer following hand washing. Epidemiol Infect 119, 319–325. 9440435 10.1017/s0950268897008261 PMC2809004

[jam14796-bib-0048] Pitt, S.J. , Crockett, S.L. and Andreou, G.M. (2018) The contribution of hand drying in prevention of transmission of microorganisms: comparison of the efficacy of three hand drying methods in the removal and distribution of microorganisms. J Infect Prev 19, 310–317. 10.1177/1757177418789485 PMC11009559 38617877

[jam14796-bib-0049] Redway, K. and Fawdar, S. (2008) A comparative study of three different hand drying methods: paper towel, warm air dryer, jet air dryer. University of Westminster, London. Available at: http://www.europeantissue.com/pdfs

[jam14796-bib-0050] Rose, J.B. and Haas, C.N. (1999) A risk assessment framework for the evaluation of skin infections and the potential impact of antibacterial soap washing. Am J Infect Control 27, S26–S33. 10586143 10.1016/s0196-6553(99)70039-8

[jam14796-bib-0051] Ryan, M.O. , Haas, C.N. , Gurian, P.L. , Gerba, C.P. , Panzl, B.M. and Rose, J.B. (2014) Application of quantitative microbial risk assessment for selection of microbial reduction targets for hard surface disinfectants. Am J Infect Control 42, 1165–1172. 25241163 10.1016/j.ajic.2014.07.024

[jam14796-bib-0052] Snelling, A.M. , Saville, T. , Stevens, D. and Beggs, C.B. (2011) Comparative evaluation of the hygienic efficacy of an ultra‐rapid hand dryer vs conventional warm air hand dryers. J Appl Microbiol 110, 19–26. 20887403 10.1111/j.1365-2672.2010.04838.x PMC3017747

[jam14796-bib-0053] Taylor, J.H. , Brown, K.L. , Toivenen, J. and Holah, J.T. (2000) A microbiological evaluation of warm air hand driers with respect to hand hygiene and the washroom environment. J Appl Microbiol 89, 910–919. 11123464 10.1046/j.1365-2672.2000.01122.x

[jam14796-bib-0054] The Dr. Oz Show (2017) The Hygienic Way to Dry Your Hands. Available at: https://www.doctoroz.com/episode/i‐told‐ya‐so‐dr‐oz‐and‐his‐wife‐lisa‐debate‐biggest‐everyday‐health‐myths?video_id=5423222426001.

[jam14796-bib-0029] The Dr. Oz Show (2018). How Dirty Are Hand Dryers? Available at: https://www.doctoroz.com/episode/dirty‐hand‐dryers‐are‐they‐germ‐breeding‐machines.

[jam14796-bib-0055] Todd, E.C.D. , Michaels, B.S. , Smith, D. , Greig, J.D. and Bartleson, C.A. (2010) Outbreaks where food workers have been implicated in the spread of foodborne disease. Part 9. washing and drying of hands to reduce microbial contamination. J Food Prot 73, 1937–1955. 21067683 10.4315/0362-028x-73.10.1937

[jam14796-bib-0056] Vermeil, T. , Peters, A. , Kilpatrick, C. , Pires, D. , Allegranzi, B. and Pittet, D. (2019) Hand hygiene in hospitals: anatomy of a revolution. J Hosp Infect 101, 383–392. 30237118 10.1016/j.jhin.2018.09.003

[jam14796-bib-0057] Vitali, F. (n.d.) The great debate: paper towels vs. air dryers. Facility Management. Available at: http://facilitymanagement.com/restroom‐paper‐towels‐air‐dryers/

[jam14796-bib-0058] Restroom Direct (n.d.) Which is better in a commercial restroom? Hand dryers or paper towels? Available at: https://www.restroomdirect.com/hand‐dryers‐vs‐paper‐towels.aspx.

[jam14796-bib-0059] Wilcox, M.H. , Best, E.L. and Parnell, P. (2017) Pilot study to determine whether microbial contamination levels in hospital washrooms are associated with hand‐drying method. J Hosp Infect 97, 201–203. 28712547 10.1016/j.jhin.2017.07.007

[jam14796-bib-0060] Wilson, A.M. , Reynolds, K.A. and Canales, R.A. (2018) Predicting viral infection risks and optimizing hygiene protocols using a modeling approach. Am J Infect Control 46, S42–S43.

[jam14796-bib-0061] Wilson, A.M. , Reynolds, K.A. , Verhougstraete, M.P. and Canales, R.A. (2019) Validation of a stochastic discrete event model predicting virus concentration on nurse hands. Risk Anal 39, 1812–1824. 30759318 10.1111/risa.13281

[jam14796-bib-0062] World Health Organization (2009) WHO Guidelines on Hand Hygiene in Health Care: First Global Patient Safety Challenge Clean Care is Safer Care. Geneva: WHO Press. 23805438

[jam14796-bib-0063] World Health Organization (n.d.) Hygiene. Available at: http://www.who.int/topics/hygiene/en/

[jam14796-bib-0064] Yamamoto, Y. , Ugai, K. and Takahashi, Y. (2005) Efficiency of hand drying for removing bacteria from washed hands: comparison of paper towel drying with warm air drying. Infect Control Hosp Epidemiol 26, 316–320. 15796287 10.1086/502546

[jam14796-bib-0065] Zivich, P.N. , Gancz, A.S. and Aiello, A.E. (2018) Effect of hand hygiene on infectious diseases in the office workplace: a systematic review. Am J Infect Control 46, 448–455. 29195781 10.1016/j.ajic.2017.10.006

